# Skeletal Site-specific Changes in Bone Mass in a Genetic Mouse Model for Human 15q11-13 Duplication Seen in Autism

**DOI:** 10.1038/s41598-017-09921-8

**Published:** 2017-08-29

**Authors:** Kirsty E. Lewis, Kunal Sharan, Toru Takumi, Vijay K. Yadav

**Affiliations:** 1 Department of Mouse and Zebrafish Genetics, Wellcome Trust Sanger Institute, Cambridge, CB10 1SA United Kingdom; 2 RIKEN Brain Science Institute (BSI), Wako, Saitama, Japan; 3 Graduate School of Biomedical Sciences, Hiroshima University, Minami, Hiroshima, Japan; 4 Metabolic Research Laboratory, National Institute of Immunology, Aruna Asaf Ali Marg, New Delhi, 110067 India; 5 Department of Physiology, Present Address: Pharmacology, Neuroscience, University of Bristol, Bristol, BS8 1TD United Kingdom; 6 Department of Molecular Nutrition, Present Address: CSIR-Central Food Technological Research Institute, Mysore, India

## Abstract

Children suffering from autism have been reported to have low bone mineral density and increased risk for fracture, yet the cellular origin of the bone phenotype remains unknown. Here we have utilized a mouse model of autism that duplicates 6.3 Mb region of chromosome 7 (*Dp*/+) corresponding to a region of chromosome 15q11-13, duplication of which is recurrent in humans to characterize the bone phenotype. Paternally inherited *Dp*/+ (*patDp*/+) mice showed expected increases in the gene expression in bone, normal postnatal growth and body weight acquisition compared to the littermate controls. Four weeks-old *patDp*/+ mice develop a low bone mass phenotype in the appendicular but not the axial skeleton compared to the littermate controls. This low bone mass in the mutant mice was secondary to a decrease in the number of osteoblasts and bone formation rate while the osteoclasts remained relatively unaffected. Further *in vitro* cell culture experiments and gene expression analysis revealed a major defect in the proliferation, differentiation and mineralization abilities of *patDp*/+ osteoblasts while osteoclast differentiation remained unchanged compared to controls. This study therefore characterizes the structural and cellular bone phenotype in a mouse model of autism that can be further utilized to investigate therapeutic avenues to treat bone fractures in children with autism.

## Introduction

The skeleton is an organ system in the body that performs many vital functions^1^. On one hand, it protects many soft organs such as brain and heart, and on the other it serves as an endocrine organ to influence function of other organs such as pancreas, testis and brain^1, 2^. Major component of the mature skeleton are bones, which are continuously remodelled, through a process known as bone remodelling, throughout life to perform these vital functions. Bone remodelling is carried out by two cell types, osteoblasts and osteoclasts^3, 4^. Osteoblasts are the mono-nucleated cells of mesenchymal origin that deposit new bone matrix while the osteoclasts are multinucleated cells of hematopoietic origin that degrade the old bone matrix^5–7^. These two cells function as a bone remodelling unit to replace the old bone matrix with a new one to maintain the mechanical strength of the skeleton. Bone remodelling is tightly regulated via a complex process, which is only now beginning to be understood^5–8^. It is affected by variety of cues, which are environmental, autocrine/endocrine, and genetic in nature. An imbalance in these cues leads to bone diseases characterized by a low bone density and an increased risk of fractures^1–8^. Many diseases with complex aetiology have long been known to be associated with low bone density and increased risk of fractures yet the underlying mechanisms remain unclear.

Autism is a neurodevelopmental disorder characterized by impaired language skills and communication, as well as repetitive and stereotypic patterns of behavior^9^. Autism is one of the most heritable psychiatric disorder suggesting that genetic factors play a large role in its development or manifestation of symptoms^10, 11^. Psychiatric symptoms in autism appear during early life, as early as 6 months of age, and persist throughout life^9^. Besides the psychiatric problems children with autism are often found to have low bone density and increased risk of fractures^12–15^. The fact that like autism, genetics and heritability plays an important role in the establishment and maintenance of bone density suggests that common factors could be involved in regulating bone density and autism^16^. However, besides genetic factors environment also plays an important role in regulating different phenotypes in autism spectrum disorders viz., physical exercise and nutrition. In the past, several knockout mice have been created to investigate different phenotypes in autism, but the molecular mechanism responsible for the pathophysiology of autism and its associated metabolic diseases is far from complete^17–25^. The above facts underscore that we need to increase our knowledge of bone development in autism in an animal model so that we can optimize dysregulated bone homeostasis in children with autism.

A genetic mouse model had been created by us earlier to investigate the psychiatric symptoms in autism following duplication of a region on the chromosome 15 i.e., 15q11.2-13.1^26^. Duplication of this chromosomal region is the recurrent cytogenetic aberration associated with autism, which occurs in up to 5% of patients^9–11, 27–33^. On the basis of conserved human/mouse linkage, a mouse model with a 6.3 Megabase (Mb) duplication of mouse chromosome 7, mirroring the human chromosome 15q11.2-13.1 duplication was created through sequential tragetting of the embryonic stem cells^26^. The paternally inherited *Dp*/+ mouse genetic model (referrred in this study as *patDp*/+ from now on) faithfully recapitulates several behavioural phenotypes observed in the autism patients and provides us an opportunity to characterize the bone phenotype in these mice. Here we report that *patDp*/+ mice display a low bone mass phentopye and charatcerise the architectural and cellular bone parameters in these mutant mice.

## Results

### Generation and analysis of mice with paternally duplicated autism loci on chromosome 7 (*patDp*/+ mice)

**Figure 1:**
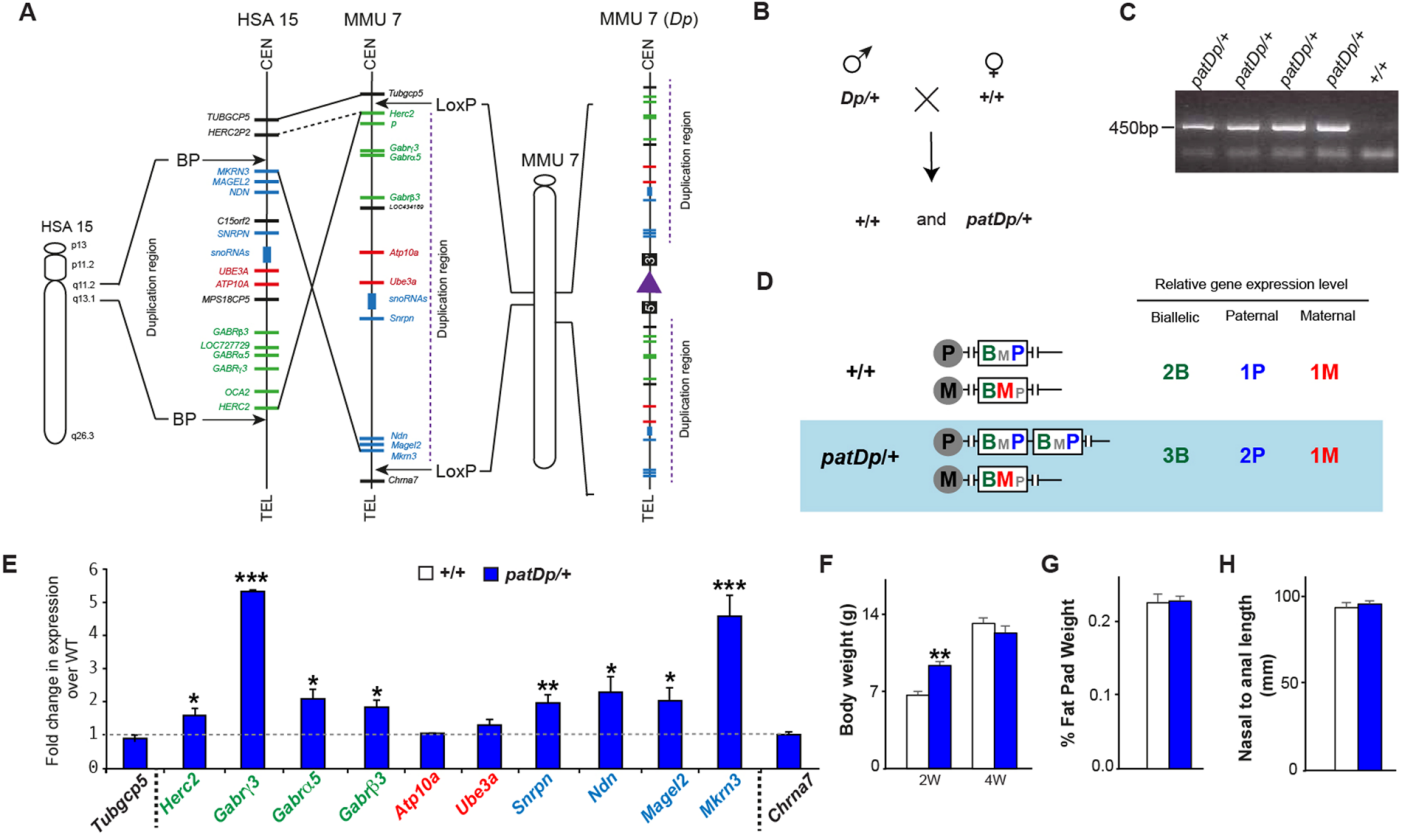
Generation and analysis of a duplicated region of chromosome 7 (*Dp*/+) in mice. (**A**) Schematic representation of the genomic regions in the human and mouse genomes showing the details of conserved linkage in human 15q11-13 and mouse chromosome 7. The duplicated region is marked by the insertional site for the 2 LoxP (Adapted from Nakatani *et al*., Cell 2009). (**B**) Schematic represetation of the breeding strategy used to generate *patDp*/+ and corresponding control WT (+/+) mice. (**C**) Genotyping PCR analysis on the tail genomic DNA to identify the +/+ and *patDp*/+ mice. The gel is the full length gel and lanes have not been cropped or stitched. (**D**) Expected expression levels in the +/+ and *patDp*/+ mice for the genes present in the duplicated region. (**E**) qPCR analysis of genes in the duplicated region and 2 genes that lie outside of the duplicated region in the long bone from +/+ and *patDp*/+ mice. Paternally expressed genes are marked with blue, maternally expressed with red and nonimprinting genes are marked with green. Dotted lines represent boundaries of the chromosomal rearrangement. (**F**–**H**) Body weight (**F**) % Fat pad weight (**G**) and nasal to anal length (**H**) analysis in +/+ and *patDp*/+ mice. n = 8–10 mice per group.

The duplication of the region of human chromosome 15q11-13 was modelled in mouse through interstitial duplication of mouse chromosome 7 corresponding to the region between common breakpoints in human chromosome 15q11-13 by chromosomal engineering (Fig. 1A) as described previously^26^. To generate the experimental cohort of mice for analysis male *patDp*/+ mice were breed with female +/+ *C57Bl6/N* mice and genotyped using PCR on the genomic DNA, these mice are labelled as *patDp*/+ mice from here on (Fig. 1B,C).

The 6.3 Mb duplication region in the *patDp*/+ mice includes the region of genomic imprinting and has the following genes: *Herc2, Gabrγ3, Gabrα5, Gabrβ3, Atp10a, Ube3a, Snrpn, Ndn, Magel2 and Mkrn3*. Because the gene expression is expected to vary depending on the tissue type, imprinting status and mode of inheritance as shown in Fig. 1D, relative gene expression was assessed by quantitative RT-PCR in the bones from *patDp*/+ mice relative to WT mice (Fig. 1E). Moreover, the expression changes in these genes in the *patDp*/+ mice has been assessed earlier in the brain and therefore it was imperative for us to establish their expression levels in the bones of these mice to further investigate their possible involvement in the cellular phenotype in the bone. In the bone, *Herc2, Gabrγ3, Gabrα5, Gabrβ3, Snrpn, Ndn, Magel2 and Mkrn3* genes in the duplicated region were increased in the expression whereas *Atp10a* and *Ube3a* did not show a significant change in the expression (Fig. 1E). As expected *Tubgcp5* and *Chrna7*, which are located outside the duplicated region, did not show any significant difference in their expression levels (Fig. 1E). *patDp*/+ mice bred normally and were fertile and as reported previously^26^ showed normal body weight compared to wild-type (WT) mice (Fig. 1F–H). *P < 0.05, **p < 0.01, ***p < 0.001.﻿

### Analysis of bone volume *and* architecture in *patDp*/+ mice

**Figure 2:**
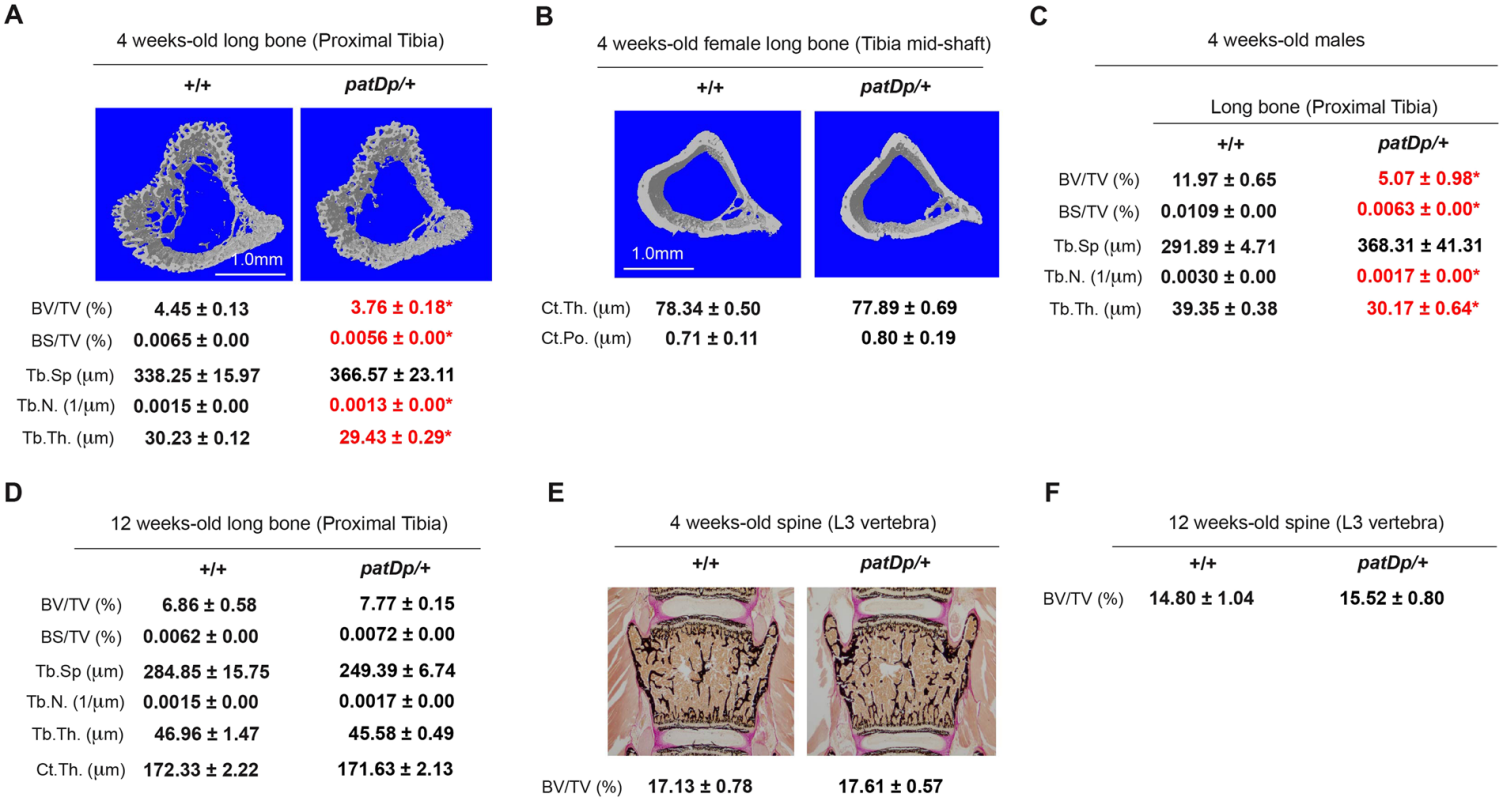
Low bone mass in the long bone but not spine of 4 week-old *patDp*/+ mice. (**A**) μCT analysis of proximal tibia in 4 week-old +/+ and *patDp*/+ mice. (**B**) μCT analysis of tibial mid-shaft in 4 weeks-old +/+ and *patDp*/+ mice. (**C**) μCT analysis of proximal tibia in 4 weeks-old +/+ and *patDp*/+ male mice. (**D**) μCT analysis of proximal tibia in 12 weeks-old +/+ and *patDp*/+ mice. (**E**,**F**) Von Kossa staining of histological sections of lumber 3 vertebra in 4 weeks (**E**)- and 12 weeks-old (**F**) +/+ and *patDp*/+ mice. *P < 0.05. Values are mean ± SEM. n = 8–10 mice were utilized per group. All mice were females except in the panel C where male mice have been used. Abbreviations used are Bone volume over total volume % (BV/TV%); Bone surface over total volume % (BS/TV%); Trabecular separation (Tb.Sp); Trabecular number (Tb.N.); Trabceular thickness (Tb.Th.); Cortical thickness (Ct.Th.) and Cortical porosity (Ct.Po.).

Having confirmed that the *patDp*/+ mice have the expected expression changes in bone for the genes present in the duplicated region we next analyzed whether significant differences in specific bone parameters exist between WT and *patDp*/+ mice. As the bone composition (cortical versus trabecular) in mammals differs depending on the skeletal site analyzed we decided to analyze two different skeletal sites, long bone and vertebra. We selected tibia as a representative long bone for μCT analysis and analyzed them at two different locations, proximal region for changes in the trabecular parameters and mid-shaft for changes in the cortical parameters. For trabecular bone analysis, we selected lumbar 4 vertebrae as a representative trabecular bone rich site. We first performed analysis on the tibia bones by μCT. As shown in Fig. 2A, the proximal tibias of 4 weeks-old female *patDp*/+ mice exhibited significant decreases in the bone volume over total volume % (BV/TV%), bone surface per total volume % (BS/TV%), trabecular number (Tb.N.) and thickness (Tb.Th.) and a tendency to have higher trabecular separation (Tb.Sp.) relative to WT littermate controls. The differences in these parameters ranged between 2.7% and 17%. We next assessed changes in cortical parameters, thickness and porosity, at tibia mid-shaft. Cortical thickness tended to be on the lower side (ns) and cortical porosity on the higher side (ns) in the *patDp*/+ relative to WT littermates (Fig. 2B). μCT analysis of 4 weeks-old male *patDp*/+ mice showed a compromised bone architecture like the female mice with significant decreases in the BV/TV%, BS/TV%, Tb.N. and Tb.Th. (Fig. 2C). The mutant animals also had a tendency towards higher Tb.Sp. (Fig. 2C). In contrast to the significant changes in the bone phenotype in the 4 weeks-old mice, 12 weeks-old *patDp*/+ mice did not display any changes in the bone architecture in tibia (Fig. 2D). We next analyzed vertebra through classical non-demineralized histology after methyl methacrylate-embedding, a standard technique used to assess vertebral histopathology. In contrast to the compromised bone structure in the tibia, analysis of vertebra collected from 4 weeks- and 12 weeks-old *patDp*/+ mice through histology and histomorphometry did not reveal any significant difference in the bone volume over total volume % between WT and mutant mice indicating that bone mass in the vertebral column is not affected in the *patDp*/+ mice (Fig. 2E,F).

### Histomorphometric analysis of 4 weeks-old WT and *patDp7*/+ female mice

**Figure 3:**
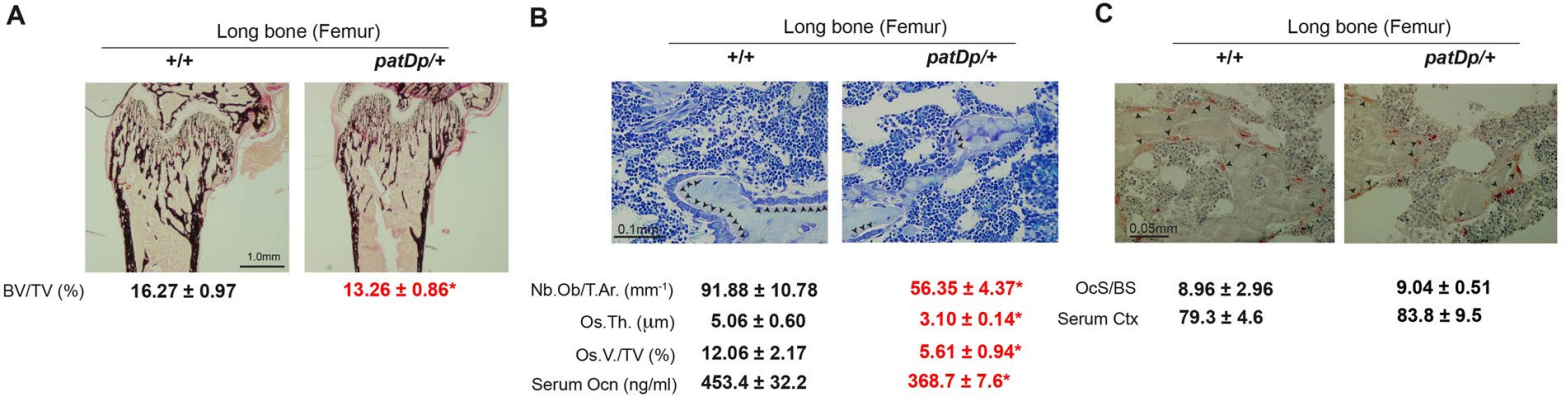
Isolated osteoblast defect contributes to the low bone mass phenotype in the long bone of *patDp7*/+ mice. (**A**) Von Kossa staining of histological section and bone volume over total volume % (BV/TV%) analysis in +/+ and *patDp*/+ mice. (**B**) Analysis of osteoblast numbers, osteoid thickness and osteoid volume per total volume % in +/+ and *patDp*/+ mice. (**C**) Analysis of osteoclast surface per bone surface and Ctx levels in 4 week-old +/+ and *patDp*/+ mice. *P < 0.05. Arrow heads indicate location of respective cells in the sections. Values are mean ± SEM. n = 8–10 mice per group. All mice were females.

Because we observed a compromised bone volume, surface and trabecular parameters in the *patDp*/+ mice in the long bones, it was of interest to further examine this bone phenotype at the cellular level. Histological and histomorphometric analysis was performed on femurs at the femoro-tibial joint of WT and *patDp*/+ mice at 4 weeks of age. This analysis, as shown in Fig. 3A, clearly demonstrates that there is a significant decrease in the trabecular bone of *patDp*/+ mice relative to WT littermates. In light of the striking defects observed in the histological analysis of femurs from female *patDp*/+ mice with regard to bone mass, we next determined if this defect was at least in part due to differences in the number of osteoblasts or osteoclasts lining the bone surface of these animals (Fig. 3B,C).

We first analyzed changes in the osteoblast numbers and function. As shown in Fig. 3B, there was a significant decline in the number of osteoblasts per trabecular area. This accounted for a significant decrease in the osteoid parameters as well. Osteoid is the protein matrix secreted by osteoblasts. Both osteoid thickness and osteoid volume/total volume % was significantly decreased in the *patDp*/+ mice relative to WT littermates (Fig. 3B). This decrease in the osteoblast numbers and function was also reflected in the serum marker of osteoblast function, osteocalcin, which was decreased by 19% in the *patDp*/+ mice (Fig. 3B). In contrast to the observed defect in the osteoblast parameters there was no significant difference in the osteoclast surface/bone surface in the *patDp*/+ mice relative to WT littermates (Fig. 3C). Accordingly, serum marker of bone resorption, collagen type 1 cross-linked C-telopeptide (Ctx) was not altered in the *patDp*/+ mice relative to WT littermates (Fig. 3C).

Overall, these findings suggest that female *patDp*/+ mice are osteopenic mainly due to a significant decrease in osteoblast numbers and function and not likely due to a large difference in the osteoclast parameters.

### Cell autonomous decrease *in* osteoblast parameters in *patDp*/+ mice

**Figure 4:**
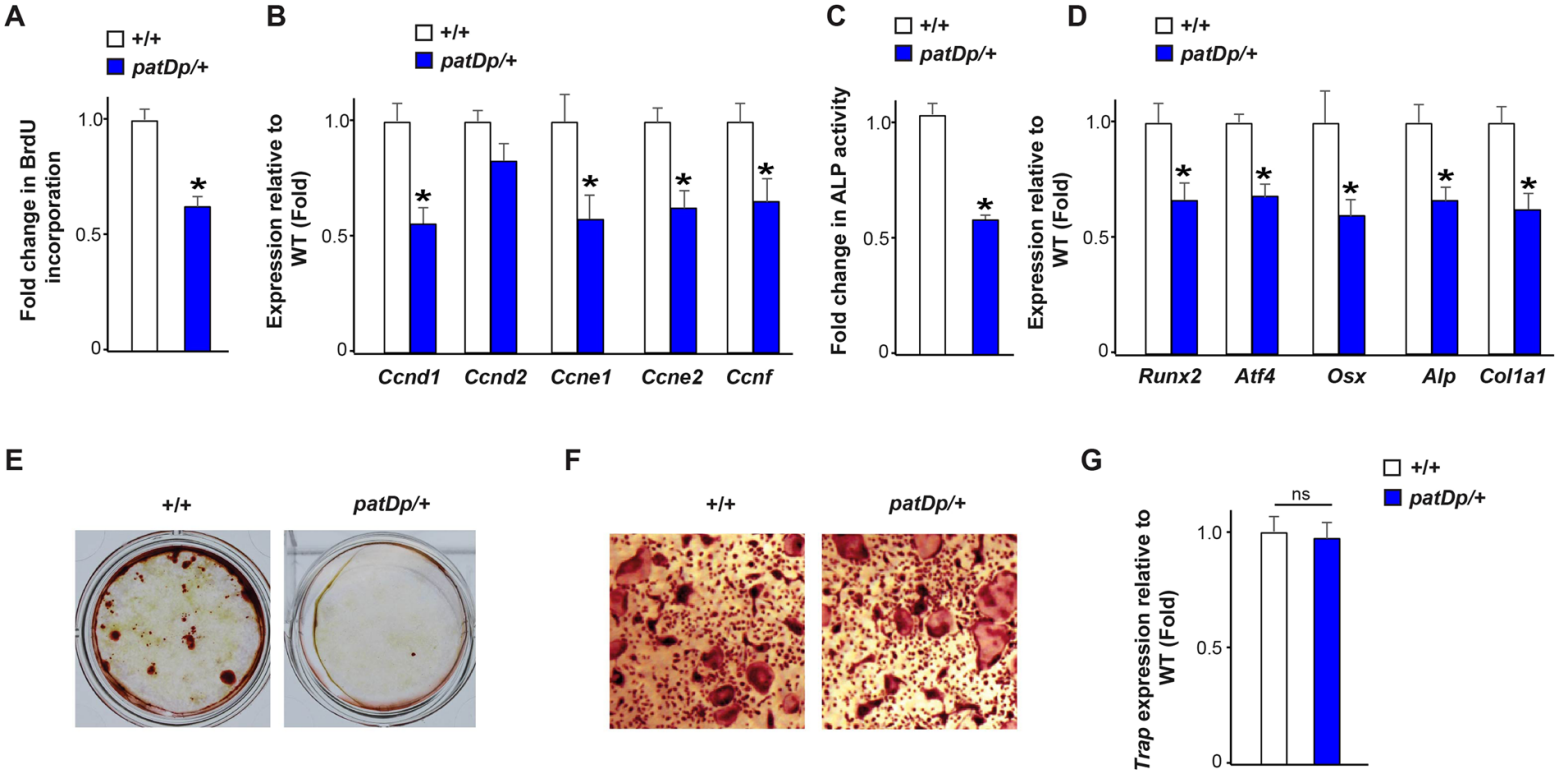
Osteoblast proliferation, differentiation and function is compromised in *patDp*/+ mice. (**A**) BrdU incorporation assay in +/+ and *patDp*/+ calvarial osteoblasts. (**B**) Real-time PCR analysis of *Cyclins* in WT and *patDp*/+ calvarial osteoblasts. (**C**) Alkaline phosphatase activity assay in +/+ and *patDp* /+ calvarial osteoblasts. (**D**) Real-time PCR analysis of osteoblast marker genes in +/+ and *patDp*/+ osteoblasts. (**E**) Alizarin red staining in +/+ and *patDp*/+ calvarial osteoblasts cultured for 21 days. (**F**,**G**) Trap staining analysis (**F**) and Trap gene expression (**G**) in differentiated osteoclasts from +/+ and *patDp*/+ mice. *P < 0.05. Values are mean ± SEM. n = 5–8 per group.

The decrease in osteoblast numbers and functions observed in the *patDp*/+ mice *in vivo* through skeletal histology and histomorphometry raises the question whether osteoblasts in these mice have a cell autonomous defect. This osteoblast defect could be either in the ability of these mutant osteoblasts to proliferate, differentiate or deposit the mineral matrix or all of the aforementioned parameters. To test this contention, we next utilized primary osteoblasts isolated from calvarias of the WT and *patDp*/+ mice and tested them for proliferation, differentiation and mineralization using *in vitro* assays (Fig. 4).

To determine if osteoblast proliferation was affected by *patDp*/+ mutation, we measured the number of cells actively synthesizing DNA by BrdU incorporation *in vitro* (Fig. 4A). Analysis of proliferation of osteoblasts isolated from WT and *patDp*/+ mice revealed a 40% decrease in proliferation of *patDp*/+ osteoblasts compared to their WT counterparts (Fig. 4A). Molecularly this decrease in proliferation of *patDp*/+ osteoblasts was associated with a decrease in the expression of *Cyclins* in these cells (Fig. 4B).

We next determined whether differentiation potential of osteoblasts was affected by *patDp*/+ mutation using two different assays, alkaline phosphatase (ALP) activity assay and marker gene expression analysis. ALP activity was decreased by ~50% in the *patDp*/+ mice osteoblasts compared to the WT counterparts (Fig. 4C). Analysis of gene expression markers of osteoblast differentiation^6^ revealed a 35–50% decrease in the expression of *Runx2*, *Atf4*, *Osx*, *Alp* and *Col1a1* (Fig. 4D).

Lastly, we looked at the effect of *patDp*/+ mutation on the osteoblasts function by analyzing changes in mineralization ability of osteoblasts isolated from the WT and *patDp*/+ mice. As shown in Fig. 4E qualitative analysis of mineralization using Alizarin red assay revealed a major decline in the mineralization potential of *patDp*/+ osteoblasts compared to the WT counterparts (Fig. 4E).

We next assayed for the ability of *patDp*/+ mutation to affect differentiation of bone marrow precursor cells into tartrate-resistant acid phosphatase (TRAP)-positive osteoclasts *in vitro*. As can be seen in Fig. 4F-G there was no significant difference in the osteoclast differentiation at the level of the multinucleated osteoclast formation (Fig. 4F) or the expression *Trap* in these cells (Fig. 4G).

Taken together these *in vitro* results demonstrate that *patDp*/+ mutation directly, and very potently, affects various aspects of osteoblast biology, but does not affect differentiation of osteoclasts.

## Discussion

In humans, it is well known that autism and autism associated disorders are often associated with a low bone density, but the bone phenotype has not been characterized in an animal model to pave a way towards future therapeutic developments to treat bone disorders in this disease^12–15^. The principal aim of our study was to characterize the bone phenotype in a mouse genetic model of autism at the structural, cellular and molecular levels.

The *Dp*/+ mice have been created with a 6.3 Mb duplication in chromosome 7 in a region that is the recurrent genetic abnormality in autism. Using a battery of tests, it has been documented that *patDp*/+ mice display many of the characteristic features of autism viz., social and anxiety abnormalities^26^. We therefore used *patDp*/+ mice for the detailed characterization of skeletal phenotype. We report that 4 weeks-old *patDp*/+ mice display structural defects in the long bones in the trabecular compartment and show a tendency towards reduced cortical integrity in the cortical compartment. This low bone mass phenotype was found to be associated with a defect in the bone formation parameters while bone resorbing cell numbers remained relatively unaltered. In contrast to changes in the long bone, bone mass was not affected in the vertebral column with the *patDp*/+ mutation. *In vitro* analysis of osteoblast and osteoclast cultures showed the *patDp*/+ mutant osteoblasts have intrinsic defects in multiple parameters compared to their WT counterparts. The above facts point towards the genes present in the *patDp*/+ region affecting the osteoblast functions through their expression in the osteoblasts.

We observed a decrease in the bone mass and architecture in the long bone and not in the vertebra of the *patDp*/+ mice whereas in humans with this disease the bone phenotype is present in both the sites^12–16^. There are important differences in the forces that determine bone deposition at different skeletal sites in mice and humans that may explain this underlying phenotypic differences observed^34, 35^. In humans both the long bones and the vertebral column are load bearing bones and show changes in bone structure and mass with changes in load^34^. In contrast in rodents the load bearing bones are only the long bones and not the vertebral column^34^. Changes in the endocrine environment and intrinsic osteoblast defects often lead to changes in both the long bones and vertebral bone mass; in contrast if the mutation in osteoblasts is affecting their ability to respond to mechanical stimuli coupled with hormonal/local changes then there is often an isolated change in bone mass in the long bone^34–36^. Our observation of a decreased bone mass in the long bone and not the vertebral column in the *patDp*/+ mice therefore points toward the intrinsic defect in *patDp*/+ osteoblasts that hampers their ability to proliferate, differentiate and mineralize and likely compromises their ability to respond to mechanical stimuli.

There are 10 genes present in the region that is duplicated in the *patDp*/+ mice and although it is difficult at the present time to point towards any particular gene ultimately responsible for the low bone mass phenotype in this model, prior studies point towards few of these genes being involved in the bone phenotype. We will now discuss the genes that may possibly be associated with a low bone mass in *patDp*/+ mice. Loss of function of *Mkrn3* has been shown to be associated with advanced bone age in humans and therefore an increase in its expression, as observed in our model, may negatively affect bone mass^37^. Mouse and human genetic studies have shown that *NECDIN* (*NDN*), *MAGEL2*, and *SNRPN* contribute to the complex neurogenetic imprinting disorder Prader–Willi-Syndrome (PWS). The symptoms of PWS, which include hyperphagia, severe obesity, hypogonadism, and behavioral abnormalities have been shown to be caused by loss of expression of these genes^38^. Based on the two PWS associated phenotypes, i.e., obesity and hypogonadism that are known to affect bone mass, few or all of these genes could be implicated in bone growth disorders. Indeed, PWS is associated with a decrease in bone density and an increased risk of fractures^37^. A decreased expression of these genes in the PWS leads to osteopenia and given that in our autism model there is ~2-fold increase in the expression of these genes, these mice would have been expected to have an increase in bone mass. In contrast to the above mentioned expected phenotype we observed osteopenia in *patDp*/+ mouse model a fact consistent with the bone phenotype observed in the autism patients. Therefore, it seems likely that effect of genes involved in the PWS is dominated by the negative effect of another gene(s) in this locus, the identity of which can only be speculated at the present time.

Three genes, *Gabrα5*, *Gabrγ3*, *Gabrβ3*, that are part of the Gamma-Aminobutyric Acid (GABA) receptor complex are most upregulated in the *patDp*/+ mice. GABA receptors mediate signals of the neurotransmitter GABA^39^. Osteoblasts constitutively express metabotropic GABA_B_ receptor subunits and not the majority of the GABA_A_ receptor subunits to which the *Gabrα5*, *Gabrγ3*, *Gabrβ3* in the *patDp*/+ locus belong^39, 40^. However, at least one of these genes, *Gabrγ3* has been shown to be induced in expression when osteoblasts are exposed to external stimuli^41^. Furthermore, it has been demonstrated that GABA can inhibit alkaline phosphatase activity and calcium accumulation in osteoblasts^42^. These evidences suggest that an increased local GABA signaling due to the observed increase in the expression of *Gabrα5*, *Gabrγ3*, *Gabrβ3* may negatively regulate the osteoblast functions in the *patDp*/+ mice. Although ours and earlier studies point toward a cell autonomous defect in the osteoblasts we cannot rule out the central signaling, perhaps through GABA, contributing towards the bone phenotype observed in the *patDp*/+ mice. Further studies are needed to identify which of the genes in the *patDp*/+ locus are expressed/induced in the osteoblasts and account for the cell autonomous decrease in osteoblast proliferation or function. This will require duplication or overexpression of genes present in this locus, either alone or in combination, to mimic the *in vivo* expression levels observed in *patDp*/+ mice.

In summary, we have shown the existence of a low bone mass phenotype in a mouse model of autism. Our results reveal an isolated decrease in osteoblast function and bone formation that accounts for this low bone mass phenotype and that osteoblasts from the *patDp*/+ mice have a cell autonomous decrease in their proliferation and differentiation. Given that autism patients often show low bone density and increased risk of fractures these results lay a foundation for development of therapies that can be used to treat bone disorders using this mouse model.

## Materials and Methods

### Animals

Studies were performed in accordance with appropriate guidelines of the Wellcome Trust Sanger Institute and the United Kingdom Home Office. All the experiments were performed under the home office project license number PPL 80/2479 and were approved by the Wellcome Trust Sanger Institute Animal Ethics Committee. Generation of *Dp*/+ mice has been described previously^26^. Heterozygous *Dp*/+ female or male mice were used for all experiments. These were generated using a *Dp*/+ male mouse and a wild type *C57Bl/6 N* female mouse and littermate controls were used for all analysis. The wild type *C57Bl/6 N* mice for breeding were obtained from in-house wild type colonies maintained at the Wellcome Trust Sanger Institute research support facility.

### Micro-computed *tomography* (µCT) analysis of long bones

Trabecular bone and cortical architecture of the proximal tibia (secondary spongiosa) was assessed using a µCT system (Skyscan 1172). Tibia bone specimen was stabilized with gauze in a 2 ml centrifuge tube filled with 70% ethanol and fastened in the specimen holder of the µCT scanner. One hundred µCT slices, corresponding to a 1.05 mm region distal from the growth plate, were acquired at an isotropic spatial resolution of 10.5 µm. A global thresholding technique was applied to binarize gray-scale µCT images where the minimum between the bone and bone marrow peaks in the voxel gray value histogram was chosen as the threshold value. The trabecular bone compartment was segmented by a semi-automatic contouring method and subjected to a model-independent morphological analysis^43^ by the standard software provided by the manufacturer of the µCT scanner. 3D morphological parameters, including model independent measures by distance transformation (DT) of bone volume fraction (BV/TV), Tb.Th. (trabecular thickness), Tb.N. (trabecular number), Tb.Sp. (trabecular separation) and connectivity density (Conn.D) were evaluated. The Conn.D is a quantitative description of the trabecular connection^44, 45^.

### Histology and *histomorphometry* of vertebra and long bone

Histological analyses were performed on femur and vertebral column specimens collected from 4 weeks-old mice using undecalcified sections stained for bone mass, osteoblasts and osteoclasts analysis as described previously^46^. Static and dynamic histomorphometric analyses were performed according to standard protocols using the Osteomeasure Analysis System (Osteometrics, Atlanta).

### Cell Cultures

#### Bromodeoxy Uridine (BrdU) cell proliferation assay

Primary calvarial osteoblasts from WT and *patDp*/+ mice at 70% to 80% confluence were trypsinized, and 5000 cells/well were seeded in 96-well plate in alpha-MEM supplemented with 10% fetal bovine serum (FBS). After 24 hours cells were left for another 24 hours in alpha-MEM supplemented with 0.5% FBS. For the last 4 hours of the 24-hour stimulation period, the cells were pulsed with BrdU. BrdU incorporation was measured using ELISA kit (Roche, IN, USA).

#### Alkaline phosphatase (ALP) activity assay

Primary calvarial osteoblasts from WT and *patDp*/+ mice at approximately 80% confluence were trypsinized, and 5000 cells/well were seeded onto 96-well plates. Cells from WT and *patDp*/+ mice were cultured for 48 hours in osteoblast differentiation medium containing alpha-MEM supplemented with 10% FBS, 10 mM beta-glycerophosphate, 50 mg/mL of ascorbic acid, and 1% penicillin/streptomycin. At the end of incubation period, total ALP activity was measured using p-nitrophenylphosphate (PNPP) as substrate, and absorbance was read at 405 nm (SensoLyte® pNPP Alkaline Phosphatase Assay kit, AnaSpec, Inc.).

#### Mineralization assay

Primary osteoblast cells were seeded onto 12-well plates (25,000 cells/well) in osteoblast differentiation medium. Osteoblasts from WT and *patDp*/+ mice were cultured for 21 days with a medium change every 48 hours. At the end of the experiment, cells were washed with PBS and fixed with 4% paraformaldehyde in PBS for 15 minutes. Alizarin red-S was used for staining mineralized nodules.

### Bioassays

Serum was prepared using BD Vacutainer® SSTTM, snap frozen in liquid nitrogen and stored at −80 °C until analyzed. Serum osteocalcin and Ctx levels were measured using ELISA assays as described previously^47^.

### Molecular studies

Total RNA was isolated from either long bone flushed of bone marrow or primary calvarial osteoblasts. Real-time PCR was performed on DNase I-treated total RNA converted to cDNA using appropriate primers and standard protocols. β-Actin amplification was used as an internal control. Genotypes of all the mice were determined by PCR. All primer sequences are listed on Supplementary Table 1.

### Statistical analysis

Results are given as mean ± standard error. Statistical Analysis was performed by Student’s t-test between two groups and one-way ANOVA followed by Newman-keuls posthoc test for more than two groups. All panels in Figs 1–4 *p < 0.05 and **p < 0.01 versus WT.

### Data availability

All the mouse strains, experimental tools and raw data present in the manuscript are available upon request.

## Electronic supplementary material

Supplementary Primer sequences
