# Supplementary material for: Skeletal Site-specific Changes in Bone Mass in a Genetic Mouse Model for Human 15q11-13 Duplication Seen in Autism
Source: Sci Rep. 2017 Aug 29;7:9902. doi: 10.1038/s41598-017-09921-8 (PMC5575059; doi:10.1038/s41598-017-09921-8)
Supplement: Supplementary file 1 — Supplementary Primer sequences [file 41598_2017_9921_MOESM1_ESM.pdf]

Supplementary information:

Skeletal Site-specific Changes in Bone Mass in a Genetic Mouse Model for Human 15q11-13 Duplication Seen in Autism

Kirsty Lewis[1$#], Kunal Sharan[1,X#], Toru Takumi[2,3] and Vijay K. Yadav[1,4*]

[1]Department of Mouse and Zebrafish Genetics, Wellcome Trust Sanger Institute, Cambridge CB10 1SA, United Kingdom

[2]RIKEN Brain Science Institute (BSI), Wako, Saitama, Japan

[3]Graduate School of Biomedical Sciences, Hiroshima University, Minami, Hiroshima, Japan

[4]Metabolic Research Laboratory, National Institute of Immunology, Aruna Asaf Ali Marg, New Delhi, India 110067

[$]Present Address: Department of Physiology, Pharmacology, Neuroscience, University of Bristol, Bristol, BS8 1TD, United Kingdom

[X]Present Address: Department of Molecular Nutrition, CSIR-Central Food Technological Research Institute, Mysore, India

[#]These authors contributed equally to this work and have been listed alphabetically

[*]Corresponding Author:

email: vijay@nii.ac.in

Supplementary Table 1

qPCR Primer sequences utilized in the study

Gene	Symbol		Sequence (5' -> 3')	Amplicon Size
Mus musculus cholinergic receptor, nicotinic, alpha polypeptide 7 (Chrna7), mRNA.	*Chrna7*	F	CACATTCCACACCAACGTCTT	106
		R	AAAAGGGAACCAGCGTACATC	
Mus musculus makorin, ring finger protein, 3 (Mkrn3), mRNA.	*Mkrn3*	F	ACAGGTGTGCATACCCCCA	101
		R	GCAGGCCCTTCTATGAGCTTC	
Mus musculus melanoma antigen, family L, 2 (Magel2), mRNA	*Magel2*	F	AGAGCGCATGTTCATTGGTG	248
		R	CCTCTACGCAGGCATAAGGAT	
Mus musculus necdin (Ndn), mRNA	*Ndn*	F	GAGGTCCCCGACTGTGAGAT	179
		R	TGCAGGATTTTAGGGTCAACATC	
Mus musculus small nuclear ribonucleoprotein N (Snrpn), transcript variant 3, mRNA.	*Snrpn*	F	TGCTACGTGGGGAGAACTTG	156
		R	CCTGGGGAATAGGTACACCTG	
Mus musculus ubiquitin protein ligase E3A (Ube3a), transcript variant 2, mRNA.	*Ube3a*	F	ATCCCAGTCTGAGGACATTGA	126
		R	GCACAAAACTCATTCGTGCAG	
Mus musculus ATPase, class V, type 10A (Atp10a), mRNA.	*Atp10a*	F	CCGCCTAGCCAATGTGTACTT	113
		R	TGACCGCCAGAATGAAGAGC	
Mus musculus gamma-aminobutyric acid (GABA) A receptor, subunit alpha 5 (Gabra5), mRNA	*Gabra5*	F	TGACCCAAACCCTCCTTGTCT	109
		R	GTGATGTTGTCATTGGTCTCGT	
Mus musculus gamma-aminobutyric acid (GABA) A receptor, subunit gamma 3 (Gabrg3), mRNA	*Gabrg3*	F	GACAGTCGCCTTCGATTCAAC	114
		R	AGCCTCCGCTGTTTTAGAATTT	
Mus musculus gamma-aminobutyric acid (GABA) A receptor, subunit beta 3 (Gabrb3), transcript variant 2, mRNA	*Gabrb3*	F	CTGCTGCCAATCTGGCTTTC	121
		R	CGTAGCCTTTCAACAGCTTGTC	
Mus musculus hect (homologous to the E6-AP (UBE3A) carboxyl terminus) domain and RCC1 (CHC1)-like domain (RLD) 2 (Herc2), mRN	*Herc2*	F	CACCTGTGTATAGAGCCAAGTCA	157
		R	TTCAACCTCAAGGCTGAGAGT	

Genotyping Primer Sequences	Symbol		Sequence (5' -> 3')	Amplicon Size
	Dup7	F	ATATGTACTTTTGCATATAGTATAC	450
		R	AGAGGAGGGCCTTACTAATTACTTA	
